# Supplementary figures and images for: Reverse Signaling by Semaphorin-6A Regulates Cellular Aggregation and Neuronal Morphology
Source: PLoS One. 2016 Jul 8;11(7):e0158686. doi: 10.1371/journal.pone.0158686 (PMC4938514; doi:10.1371/journal.pone.0158686)

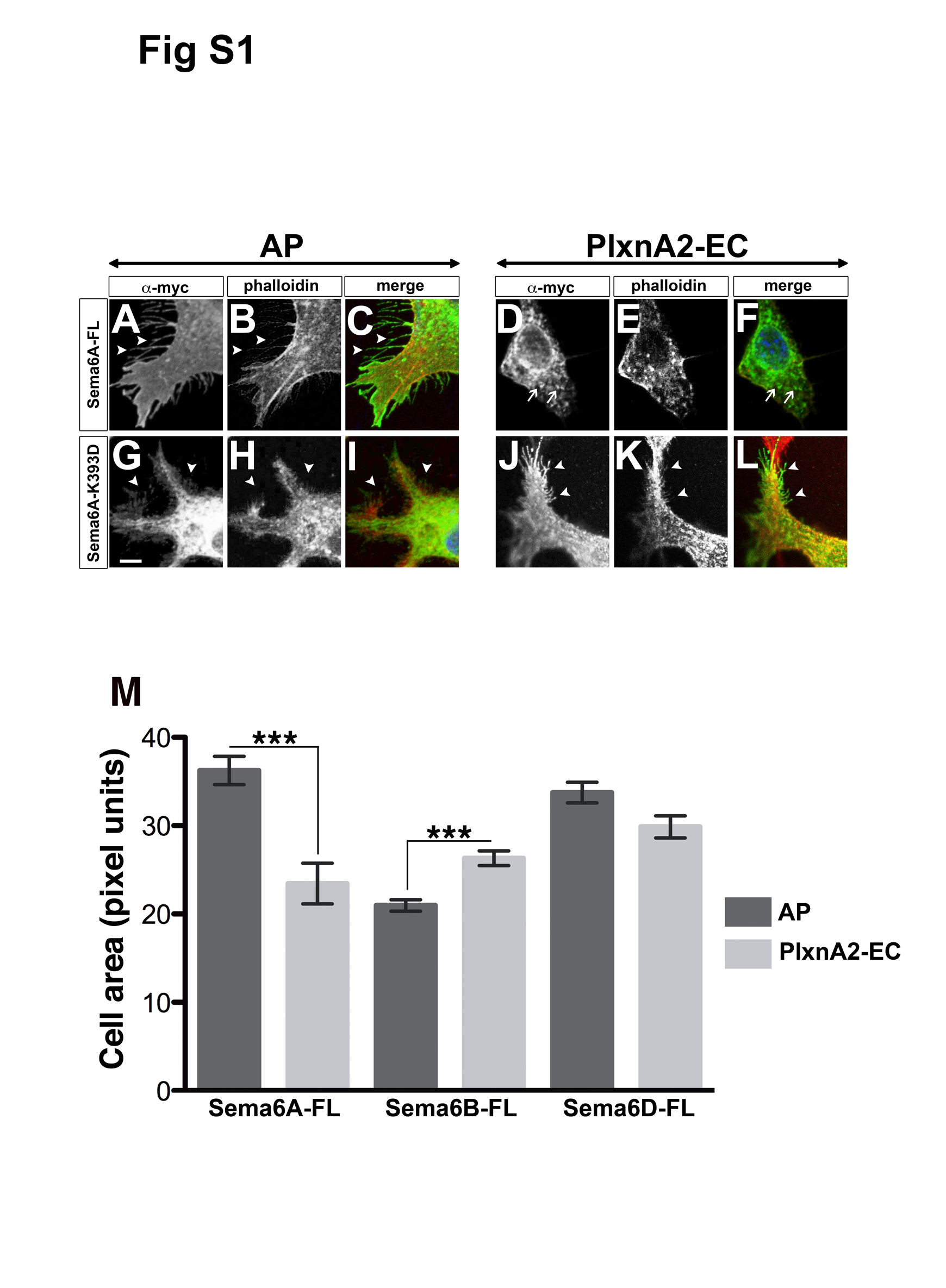

Supplement: S1 Fig — (A-L) NIH3T3 cells expressing myc-Sema6A-FL (Sema6A-FL; A-F), or myc-Sema6A-K393D (Sema6A-K393D; G-L) were treated with purified AP-Fc (AP; A-C and G-L) or PlxnA2-EC-Fc (PlxnA2-EC; D-F and J-L) and stained with anti-myc antibody and phalloidin to visualise actin fibres. Arrowheads indicate membrane protrusions, and arrows point to collapsed actin clumps. Scale bar = 5 μm. (M) Graph represents the cell area in NIH3T3 transfected with Sema6A-FL, Sema6B-FL or Sema6D-FL and treated with AP or PlxnA2-EC; n = 100–200 cells per experimental condition. Data are expressed as mean ± s.e.m; ***P <0.001; Student’s t-test. (TIF) [file pone.0158686.s001.tif]

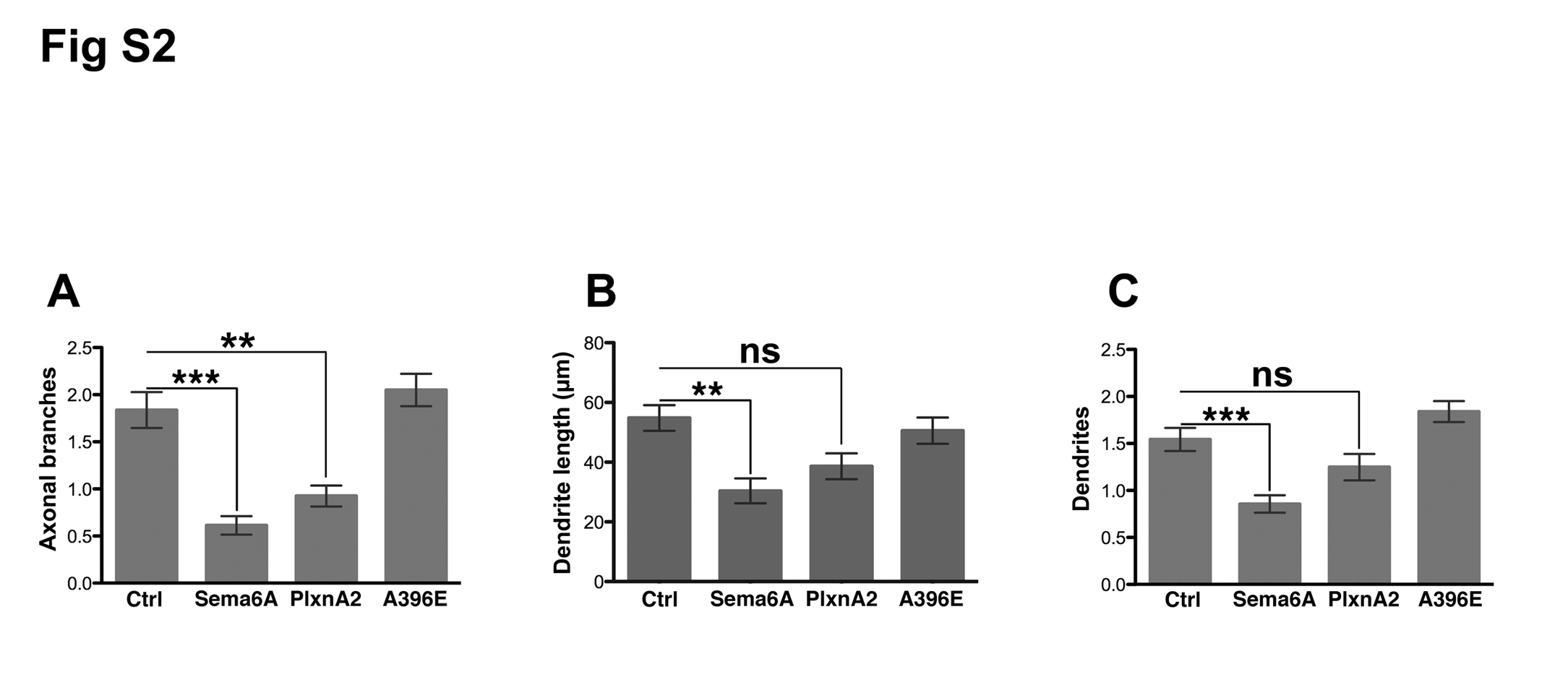

Supplement: S2 Fig — (A) Graph represents the number of axonal branches of granular neurons cultured on different NIH3T3 layers. (B) Graph represents the dendrite length of granular neurons cultured on different NIH3T3 layers. (C) Graph represents the number of dendrites per neuron in granular neurons cultured on different NIH3T3 layers; n = 100–200 cells per experimental condition. Data are expressed as mean ± s.e.m; **P ≤0.005, ***P < 0.001 and ns = P > 0.05; one-way ANOVA followed by Bonferroni multiple comparison test. (TIF) [file pone.0158686.s002.tif]

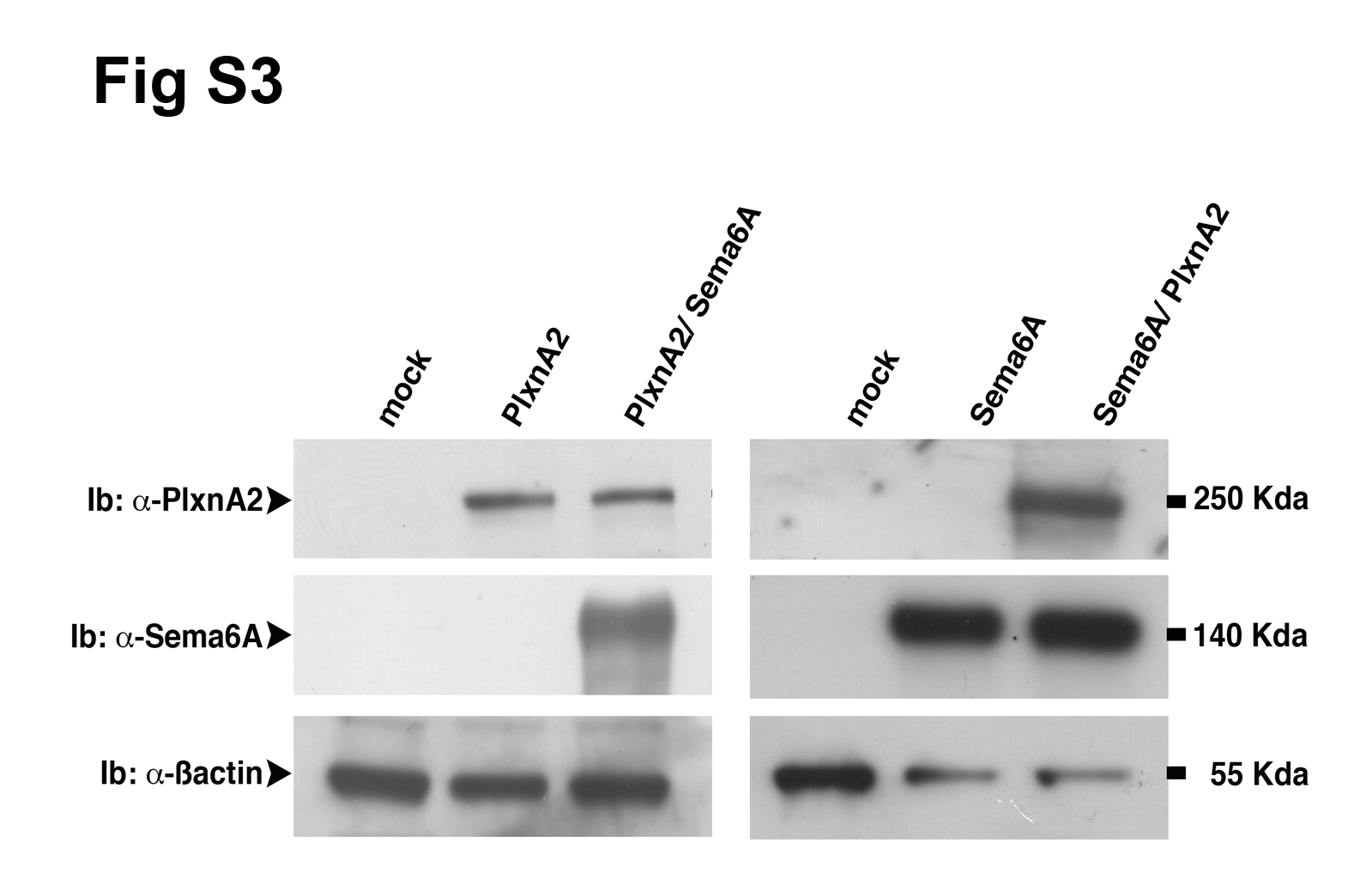

Supplement: S3 Fig — Immunoblots of total lysates from transfected COS-7 cells expressing PlxnA2, Sema6A or PlxnA2 and Sema6A shows roughly comparable levels of expression of the two proteins and no change in expression levels when they are co-expressed. (TIF) [file pone.0158686.s003.tif]

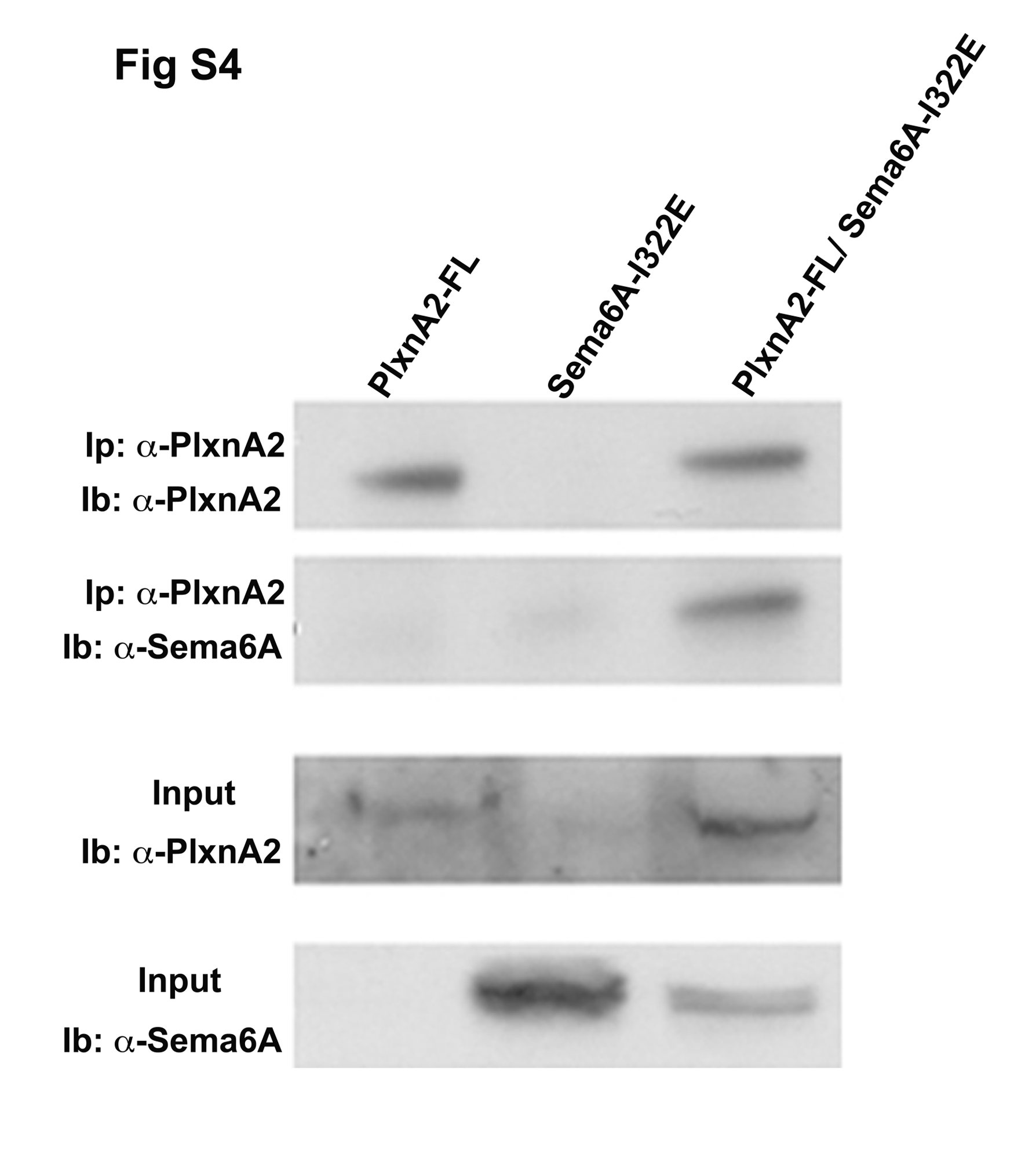

Supplement: S4 Fig — PlxnA2 immunoprecipitations (Ip) from protein samples of COS-7 cells transfected with PlxnA2-FL, Sema6A-I322E or PlxnA2-FL together with Sema6A-I322E. Antibodies against PlxnA2 and Sema6A were used in the immunoblots (Ib). (TIF) [file pone.0158686.s004.tif]

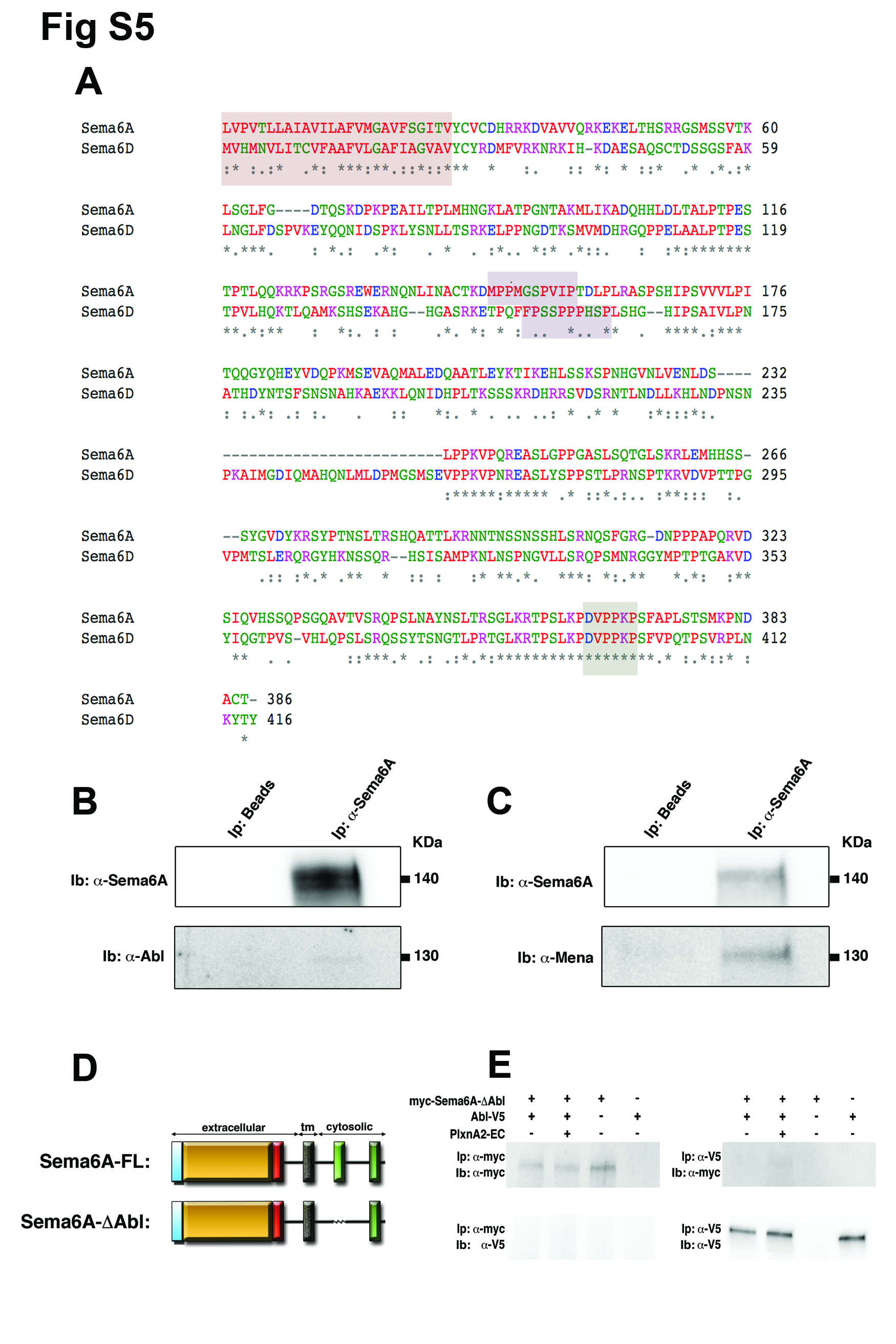

Supplement: S5 Fig — (A) Alignment of the cytosolic domains of Sema6A and Sema6D. Orange outline = transmembrane domain; purple outline = Abl binding domain; green outline = Evl binding domain. (B) Abl protein is co-immunoprecipitated with anti-Sema6A antibodies from extracts of P6 cerebellum (top); control blot with anti-Sema6A (bottom). (C) Mena protein (at ~95kDa) is also co-immunoprecipitated with anti-Sema6A antibodies from extracts of P6 cerebellum (top); control blot with anti-Sema6A (bottom). (D) Scheme indicates the structure of Sema6A-FL and Sema6A-∆Abl. (E) Immunoprecipitations from untreated or PlxnA2-EC-treated COS-7 cells transfected with different combinations of myc-Sema6A, Abl-V5. Immunoprecipitations (Ip) and Immunoblots (Ib) were performed employing anti-myc and anti-V5 antibodies. (TIF) [file pone.0158686.s005.tif]

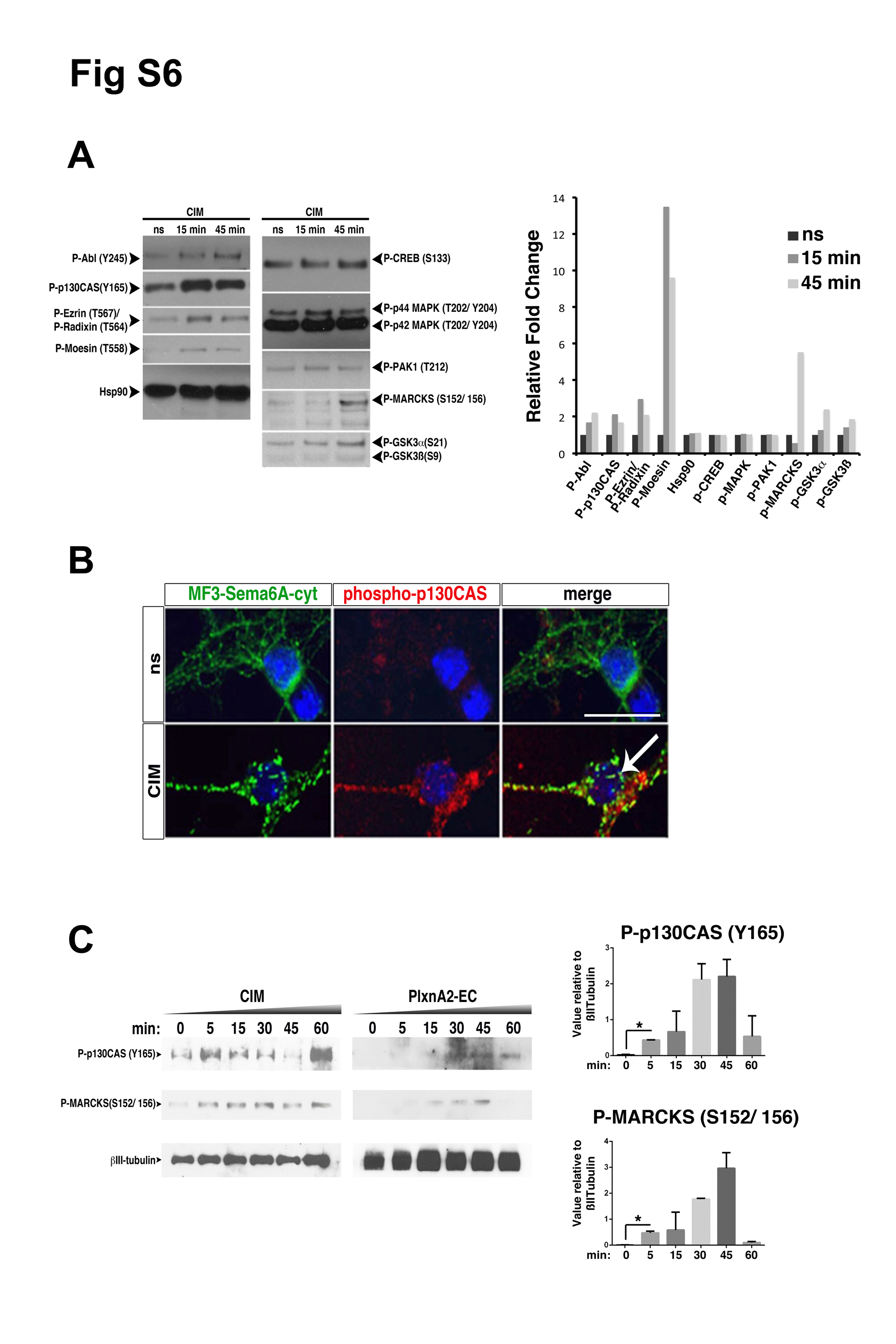

Supplement: S6 Fig — (A) Representative immunoblots from MF3-Sema6A-cyt-expressing cerebellar neurons treated with CIM for different lengths of time (min). The phosphorylation of diverse signaling cues was tested by immunoblotting. Hsp90 was used as loading control. Graph shows relative fold increase of phosphorylated proteins over the two time-points. (B) The clustering of Sema6A-cyt induces the phosphorylation of p130CAS. Moreover, Sema6Acyt clusters co-localised with anti-P-p130Cas signal (arrow). Scale bar = 20 μm. (C) Immunoblots from MF3-Sema6A-cyt-expressing cerebellar neurons treated with CIM, and cerebellar neurons treated with PlxnA2-EC for different lengths of time (min). The phosphorylation of p130CAS and MARCKS were evaluated with specific phospho-antibodies. βIII-tubulin was used as loading control. Graphs show quantification of the levels of P-p130Cas and P-MARCKS to PlxnA2-EC over multiple time-points from two replicates. Data are expressed as mean ± s.e.m; *P ≤0.05; one-way ANOVA followed by Bonferroni multiple comparison test. (TIF) [file pone.0158686.s006.tif]
